# Observation of laser-induced electronic structure in oriented polyatomic molecules

**DOI:** 10.1038/ncomms8039

**Published:** 2015-05-05

**Authors:** P. M. Kraus, O. I. Tolstikhin, D. Baykusheva, A. Rupenyan, J. Schneider, C. Z. Bisgaard, T. Morishita, F. Jensen, L. B. Madsen, H. J. Wörner

**Affiliations:** 1 Laboratorium für Physikalische Chemie, ETH Zürich, 8093 Zürich, Switzerland; 2 Moscow Institute of Physics and Technology, Dolgoprudny 141700, Russia; 3 FOSS Analytical A/S, FOSS Allé 1, Hillerød DK-3400, Denmark; 4 Department of Engineering Science, The University of Electro-Communications, 1-5-1 Chofu-ga-oka,Chofu-shi, Tokyo 182-8585, Japan; 5 Department of Chemistry, Aarhus University, Aarhus C 8000, Denmark; 6 Department of Physics and Astronomy, Aarhus University, Aarhus C 8000, Denmark

## Abstract

All attosecond time-resolved measurements have so far relied on the use of intense near-infrared laser pulses. In particular, attosecond streaking, laser-induced electron diffraction and high-harmonic generation all make use of non-perturbative light–matter interactions. Remarkably, the effect of the strong laser field on the studied sample has often been neglected in previous studies. Here we use high-harmonic spectroscopy to measure laser-induced modifications of the electronic structure of molecules. We study high-harmonic spectra of spatially oriented CH_3_F and CH_3_Br as generic examples of polar polyatomic molecules. We accurately measure intensity ratios of even and odd-harmonic orders, and of the emission from aligned and unaligned molecules. We show that these robust observables reveal a substantial modification of the molecular electronic structure by the external laser field. Our insights offer new challenges and opportunities for a range of emerging strong-field attosecond spectroscopies.

## Abstract (web summary)

Although expected to have an influence, the effect of strong laser fields on molecules in high-harmonic generation in gases is rarely explored. Kraus *et al.* show that the laser modifies the electronic structure of large polyatomic molecules and present a new theoretical framework to model this.

## Graphical abstract

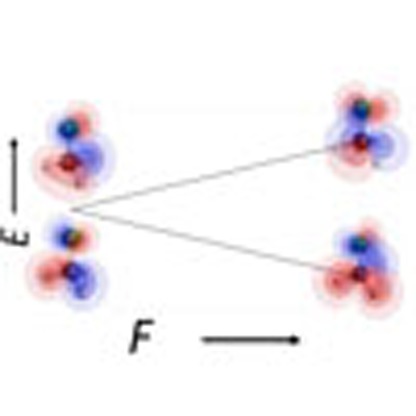

Attosecond and few-cycle laser pulses offer new opportunities for studying the electronic structure and dynamics of molecules (see, for example, refs 1, 2, 3, 4, 5, 6, 7). High-harmonic spectroscopy (HHS), on which we focus here, relies on the laser-driven recombination of an electron with its parent cation that characterizes both the electronic structure and dynamics of the molecule. The photorecombination step accesses the richness of electronic-structure information previously studied over decades by photoelectron and photoionization spectroscopy with the important additional benefit of temporal resolution.

An essential difference between high-harmonic and photoionization spectroscopies is the presence of a strong laser field in the case of HHS. This field could thus be expected to dynamically modify the electronic structure of the target. However, no such effects have been reported to date and therefore HHS has been extensively used for obtaining field-free electronic-structure information^8^,^9^. This working hypothesis was justified by solving the time-dependent Schrödinger equation for atoms exposed to intense laser pulses and comparing the results with photorecombination cross-sections of the parent ion^8^,^10^. Examples of electronic-structure effects studied by HHS include Cooper minima^9^,^11^,^12^,^13^, giant resonances^14^ and shape resonances^15^,^16^. Basic quantum-mechanical considerations reveal that the similarity between photoionization and high-harmonic spectroscopies cannot extend to large molecules because of their spatial extension and high polarizabilities.

In this article, we show how a strong laser field modifies the electronic structure of molecules and how to measure it. We chose methyl fluoride (CH_3_F) and methyl bromide (CH_3_Br) as examples because their large permanent dipole moments result in a strong, first-order coupling to the laser electric field. The laser field is found to lift the degeneracy of the electronic ground state of the molecular cation (of E-symmetry in the C_3v_ point group) and to reshape the structure of its two wave-function components. This laser-induced modification of the electronic structure of the molecules is verified experimentally by accurately measuring high-harmonic spectra of aligned and oriented molecules. We determine intensity ratios between even and odd harmonics and between different alignment distributions. These ratios are found to be robust with respect to intensity and wavelength of the driving laser pulses. Our experimental observations are shown to strongly contrast with the prediction of the state-of-the-art theory of HHS that relies on field-free models of photorecombination^8^,^9^,^17^. We therefore develop a theory that consistently includes the laser field in all three steps of HHS: ionization, propagation and recombination. The model reproduces the experimental observations with quantitative accuracy. Our study reveals new opportunities for HHS to precisely characterize the electronic structure of molecules in intense laser fields. Finally, we also show that the observables of our experiment distinguish between different sets of linear combinations of a degenerate electronic state, which is impossible in the absence of an external field.

## Results

### Experimental results

We use impulsive orientation and/or alignment to characterize the electronic structure of the molecules. By varying the time delay between the non-resonant orientation and high-harmonic-generation pulses, we probe the orientation-dependent high-harmonic yield in the time domain. Since the optimal laser parameters for orientation and alignment differ slightly, we report two sets of measurements. In the first experiment, we impulsively align the molecules using temporally stretched (150 fs) one-colour (800 nm) laser pulses at non-ionizing intensities. In the second experiment, we impulsively orient the molecules using shorter (80  fs) two-colour (800+400 nm) laser pulses. The molecules CH_3_F or CH_3_Br are diluted to 10% in a supersonic expansion in helium. Further details are given in the Methods section. In both experiments, we measure high-order harmonic spectra around the first full rotational revival of the molecules using laser pulses centred at 800, 1,275 or 1,330 nm and multiple intensities. The probe-laser parameters are identical in the alignment and orientation experiments.

**Figure 1:**
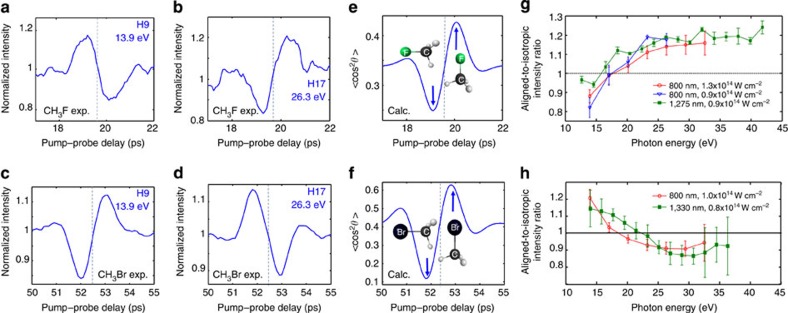
High-harmonic emission from aligned CH_**3**_**F and CH**_**3**_**Br molecules.** Measured modulation of emission intensity in the ninth harmonic (H9) or H17 of an 800-nm driving field from CH_3_F (**a**,**b**) and CH_3_Br (**c,d**). (**e**,**f**) Calculated value of the alignment parameter 〈cos^2^ *θ*〉 for CH_3_F and CH_3_Br using the experimental conditions (see Methods). The vertical lines in **a**–**f** mark the time corresponding to the classical period of rotation of the C–*X* axis. (**g**) Measured intensity ratio of emission from aligned (pump–probe delay of 20.1 ps) divided by isotropic molecules measured at different wavelengths and intensities. (**h**) Same as **g** for CH_3_Br. The error bars correspond to twice the s.d. of the measured signal fluctuations.

Figure 1 shows the results of the impulsive alignment experiment. The variations of the high-harmonic signal generated by an 800-nm probe pulse in the vicinity of the first full rotational revival are shown in Fig. 1a–d. Low- and high-harmonic orders display opposite modulations of the emitted intensity. The molecules CH_3_F and CH_3_Br also display opposite modulations with respect to each other. These results can be related to the orientation dependence of high-harmonic emission by comparing them to the degree of axis alignment (〈cos^2^*θ*〉), shown in Fig. 1e,f calculated for our experimental conditions^18^. The degree of alignment first goes through a minimum when the C–*X* (*X*=F, Br) axis is aligned perpendicular to the laser field (anti-alignment), then through a maximum when the C–*X* axis lies parallel to the laser field (alignment). Hence, the low-order harmonics of CH_3_F (for example, ninth harmonic (H9)) and the high-order harmonics of CH_3_Br (for example, H17) are most intense when the C–*X* axis is close to perpendicular to the laser field. The high-order harmonics of CH_3_F (for example, H17) and the low-order harmonics of CH_3_Br (for example, H9) are most intense when the C–*X* axis is close to parallel to the laser field.

This harmonic signal modulation is further quantified by the intensity ratio of aligned to isotropically distributed molecules, the ‘aligned-to-isotropic ratio'. This quantity, measured in multiple experiments using different driving laser intensities and central wavelengths, is displayed in Fig. 1g,h for CH_3_F and CH_3_Br, respectively. The ratios are found to cross unity between 16 and 18 eV in the case of CH_3_F and between 18 and 21 eV in the case of CH_3_Br. The reversal of the modulation is found to be independent of both the wavelength and the intensity of the probe pulse within the accuracy of the measurements. This excludes interference between the emission involving multiple electronic states of the cation as the origin of the observed revival inversion^19^,^20^.

**Figure 2:**
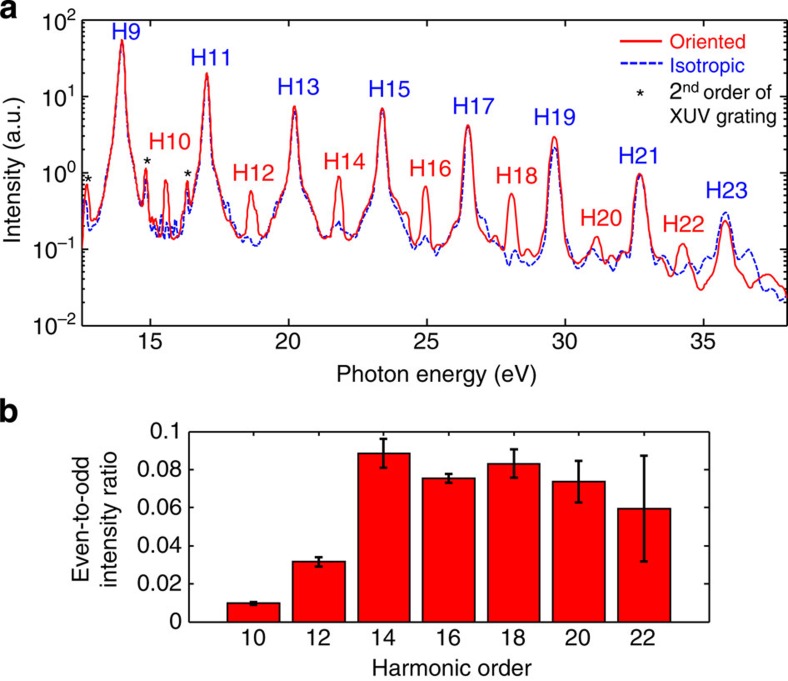
High-harmonic spectra of oriented CH_**3**_**F molecules.** (**a**) High-harmonic spectrum of CH_3_F molecules recorded at the instant of maximal orientation, that is, a delay of 19.5 ps between the two-colour pump and the 800-nm probe pulses (full red line) or for a nearly isotropic distribution (20.5 ps, dashed blue line). (**b**) Even-to-odd intensity ratio (2 × *I*_2*n*_/(*I*_2*n*−1_+*I*_2*n*+1_)) at the instant of maximal orientation, that is, a delay of 19.5 ps. The error bars correspond to twice the s.d. of the measured signal fluctuations.

Figure 2a shows the high-harmonic spectrum of oriented CH_3_F molecules recorded at the delay of maximal orientation (19.5 ps) between the two-colour pump and the 800-nm probe pulse. Besides the appearance of the commonly observed odd-harmonic orders, even harmonics are also emitted, demonstrating the orientation of the molecular sample^21^. Remarkably, the intensities of the even-harmonic orders follow a different envelope than those of the odd-harmonic orders. This observation is further quantified by the intensity ratio of the even-harmonic orders (2 × *I*_2*n*_) to the average of their two adjacent odd-harmonic orders (*I*_2*n*−1_+*I*_2*n*+1_), which is shown in Fig. 2b (the ‘even-to-odd ratio'). This ratio displays a sharp increase at low orders until H14 (22 eV) where a local maximum is observed. No even-harmonic emission was observed from CH_3_Br under the same experimental conditions. Independent Coulomb-explosion experiments showed that CH_3_Br orients very weakly under the action of a femtosecond two-colour laser field (Ram *et al.*, (manuscript in preparation)).

### Theoretical results

In the following, we introduce a theory that accounts for the influence of the laser field on the electronic structure of the target molecule in all three steps of high-harmonic generation. We then use this theory to demonstrate the substantial manifestations of laser-induced electronic-structure effects in high-harmonic spectra. Our theoretical work builds on previous results that have hinted at the role of the laser field in strong-field spectroscopies. Laser-induced mixing of multiple electronic states was predicted to modify the angle dependence of strong-field ionization (SFI)^22^. Different Stark shifts of the neutral and ionic species substantially modify the angle-dependent SFI rates of polar molecules^23^,^24^,^25^. The same effect has also been predicted^26^ and recently observed^16^ to modify the phase of the emitted high-harmonic signal. Finally, field distortions of the electronic states entering the photorecombination dipole moments were predicted to modify the emitted harmonic spectra^27^,^28^. However, to the best of our knowledge, no direct experimental evidence of this effect has been reported yet.

**Figure 3:**
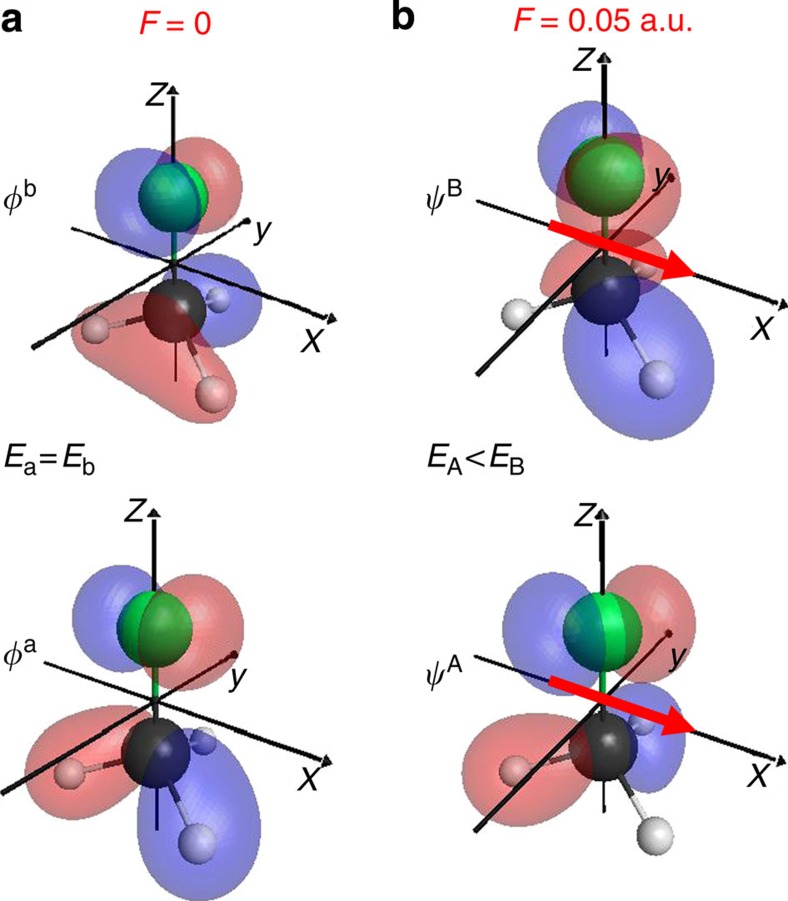
Effect of an electric field on the electronic structure of CH_**3**_**F.** The two components of the degenerate highest occupied molecular orbital (HOMO) of CH_3_F from a HF//aug-cc-pVTZ calculation in the absence (**a**) or presence (**b**) of a static electric field of amplitude 0.05 a.u. (corresponding to a laser peak intensity of 0.88 × 10^14^ W cm^−2^, isocontour value 0.1).

The 
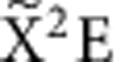
 electronic ground state of CH_3_F^+^ is well described by a one-hole configuration with an electron removed from the highest occupied molecular orbital (HOMO) of E-symmetry. Consequently, we will use the term ‘orbitals' to refer to the canonical Hartree–Fock orbitals of the neutral molecule instead of equivalently referring to the *N*−1 electron wave functions of the cation. Figure 3 shows the effect of an applied static laser field on the two components of the HOMO. The corresponding Dyson orbitals are virtually indistinguishable from the Hartree–Fock orbitals and are shown in the Supplementary Fig. 1.

**Figure 4:**
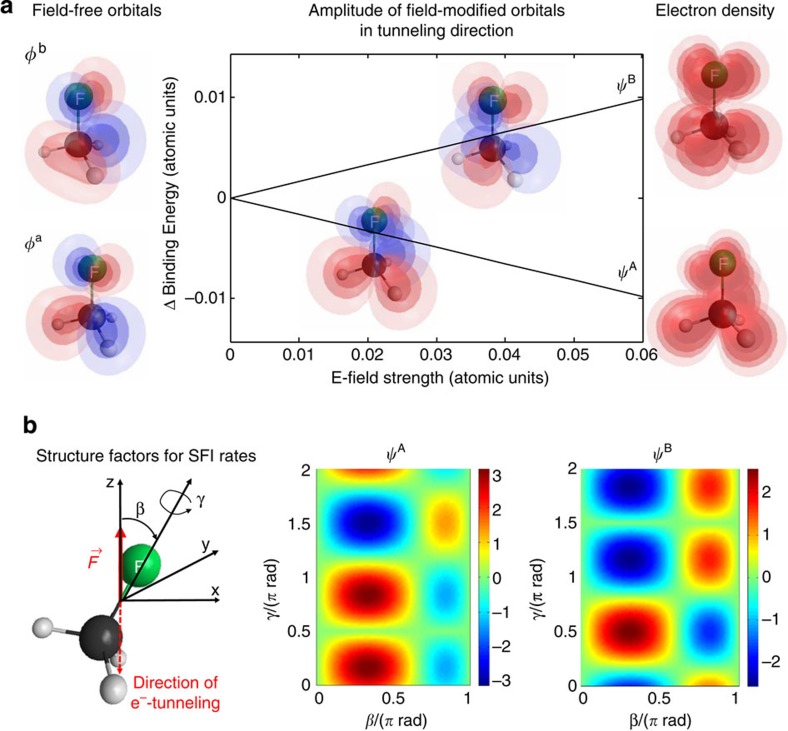
Laser-induced electronic-structure effects. (**a**) Linear Stark effect of the HOMO of CH_3_F for *β*=*π*/2. The displayed electronic eigenfunctions in a static electric field 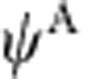 and 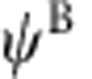 correspond to linear combinations of the two field-free components *φ*^a^ and *φ*^b^ of the HOMO (see Equation (2)). The colours encode the relative sign of the wave functions. (**b**) Structure factors 
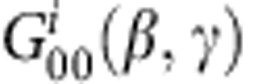
 (see Equation (4)) determining the strong-field ionization rates obtained using the weak-field asymptotic theory for the strong-field ionization of two field-mixed components of the HOMO.

The interaction of a molecule with an external electric field is given by the operator 
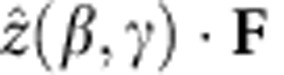
, where **F** is the electric field of amplitude *F*, *β* and *γ* are the Euler angles describing the rotation of the molecule-fixed axis system relative to the laboratory frame^29^,^30^ as shown in Fig. 4b, and 
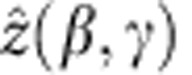
 is a 2 × 2 matrix in the basis (*φ*^a^ and *φ*^b^) of the two degenerate components of the field-free HOMO. The eigenvalues of this matrix are:


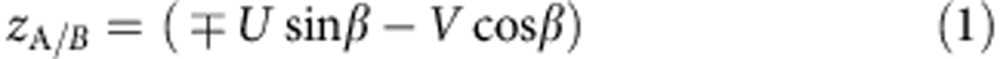


where *U* and *V* are coupling constants obtained from quantum chemical calculations with applied static electric fields (see Methods section). The corresponding eigenfunctions 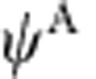 and 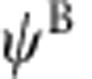 are given by:


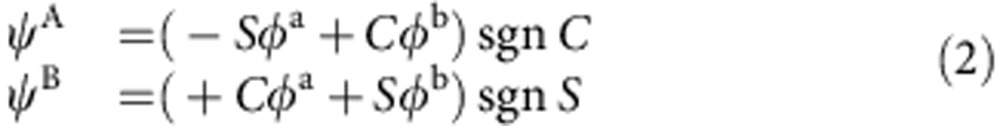


where *S*=sin(*γ*/2+*π*/4) and *C*=cos(*γ*/2+*π*/4). The Stark effect and the corresponding field-induced orbital mixing is illustrated in Fig. 4a, where the changes in the binding energies of 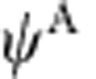 and 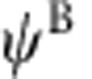 are displayed for *β*=*π*/2. Instead of the eigenfunctions that would look similar to Fig. 3b, we show isocontour representations of the eigenfunction amplitudes corresponding to particular values of *γ*. For each orientation of the molecules relative to the laser polarization, the laser field selects a particular *γ*-dependent linear combination of the field-free basis as new eigenfunctions and lifts the degeneracy (except for *β*=0 and *π*). Remarkably, the Stark shift of the binding energies depends only on *β*, while the mixing coefficients *S* and *C* depend only on *γ*. While this property is specific to the *C*_3*v*_ point group, the field effects described here are general for symmetric-top molecules and are easily extended to arbitrary asymmetric-top molecules.

We calculate the angular variation of the SFI rates of CH_3_F and CH_3_Br within the adiabatic theory^31^ because SFI of these molecules under the present experimental conditions is expected to be well described by the quasi-static approximation. More specifically, the weak-field asymptotic theory (WFAT) of tunnelling ionization introduced in ref. 29 and applied to linear^30^ and non-linear^32^ molecules is generalised to degenerate orbitals of non-linear molecules and to incorporate the linear Stark effect described in the preceding paragraph. The WFAT describes SFI of molecules as a multichannel eigenvalue problem in parabolic coordinates (*ξ*,*η*,*φ*). Within the WFAT the leading-order term in the ionization rate, Γ=|*G*_00_(*β*,*γ*)|^2^*W*_00_(*F*), factorizes into the structure factor *G*_00_(*β*,*γ*) and the field factor *W*_00_(*F*). The indices *n*_*ξ*_=0 and *m*=0 of both quantities are the parabolic quantum numbers of the dominant ionization channel. The structure factor *G*_00_(*β*,*γ*) depends on the molecular orbital, the associated Stark shift and the orientation of the molecule relative to the probe field. Its calculation is described in the following paragraph. We note that this factor also includes the sign of the asymptotic orbital wave function (colour coded in Fig. 4b), which accounts for the effect of the nodal structure of the orbitals on the orientation-dependent phase of the continuum electron wave packet^29^,^31^. This result of the WFAT for molecules was also found to be essential for modelling high-harmonic spectra of polyatomic molecules^33^. The field factor is given by


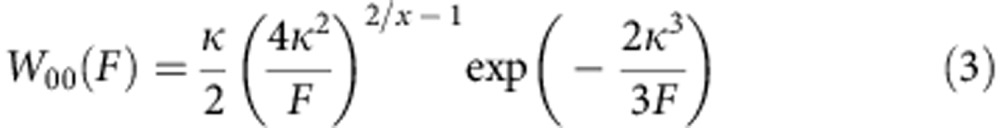


where 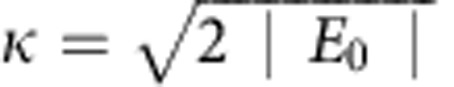, *E*_0_ is the field-free energy of the degenerate HOMOs.

An accurate calculation of the structure factors requires orbital wave functions with precise long-range tails that are very difficult to obtain in standard quantum chemistry methods. We therefore use specifically designed basis sets and variationally optimize the basis-set exponents to minimize the Hartree–Fock energy of the HOMO^34^. This procedure is applied to basis sets of five different quality levels ranging from unpolarized double to polarized quintuple zeta quality (pc-*n*, *n*=0–4). Further details are given in the Methods section. The structure factor, 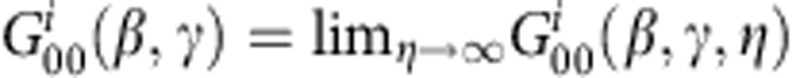, for each of the two components of the field-modified orbitals *i*=A, B is then calculated from the asymptotic value of the structure function,


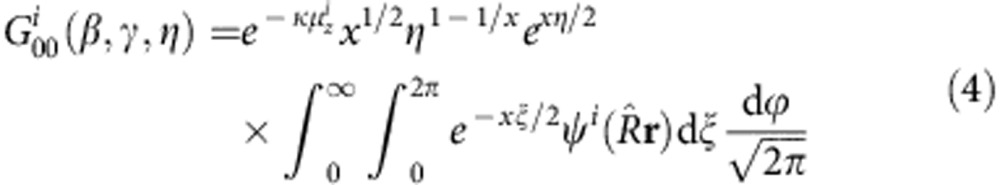


Here 
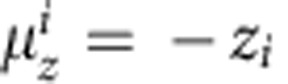
 is the *z*-component of the dipole of 
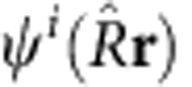
, where *z*_*i*_ is defined in Equation (1), and 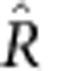 describes the rotation from the laboratory frame to the molecular frame. The extraction of the structure factor from the structure function proceeds as discussed in ref. 34 with additional details given in ref. 32: 
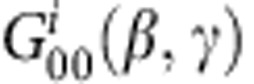
 is determined at the maximal *η* where the results of pc-(*n*−1) and pc-*n* coincide. The squared structure factors of the field-free and field-modified orbitals are shown in the Supplementary Fig. 2.

Within our theory, SFI thus prepares the eigenstates of the instantaneous Hamiltonian while a possible coupling between the two one-hole states occurs only in second order in the field and can be neglected. Consequently, the electron hole remains in the orbital in which it was created from the moment of ionization until the electron recombines. SFI is found to preferentially occur from the orbital with the largest dipole moment (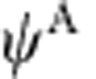), which corresponds to an electron density characterized by maxima in the F–C–H planes.

The contribution to the phase of the emitted radiation originating from the propagation step is evaluated from the quasi-classical action 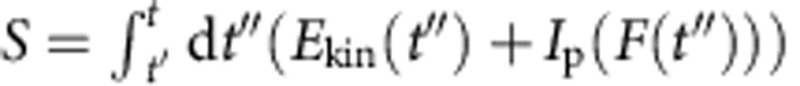
35. Since the laser field does not add a channel-specific contribution to the kinetic energy *E*_kin_(*t*′′) of the continuum electron, we include the field as a first-order correction to the *I*_p_-dependent action for each trajectory^26^.


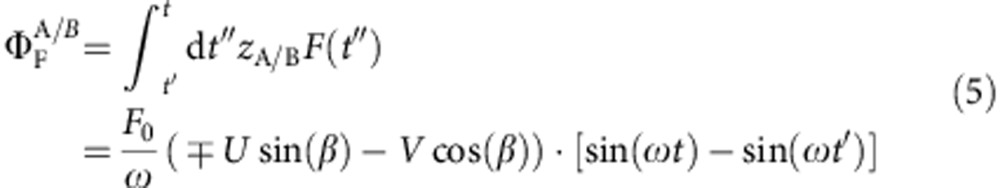


where the long-pulse limit is used and *F*_0_ is the peak electric-field amplitude. *t* and *t*′ represent the harmonic-order-specific return and birth times, respectively. The calculation of the molecular-frame photorecombination dipoles *d*_rec_(Ω,*β*,*γ*) corresponding to the observed harmonic frequencies Ω is described in the Methods section. The effect of the laser field is taken into account by transforming the photorecombination dipole moments according to Equation (2).

This theory now allows us to calculate the induced dipole moment as product of the contributions from each step involved in the process of high-harmonic generation. We coherently average the single-molecule induced dipole moment, that is, the product of the ionization, propagation and recombination amplitudes, over the molecular axis distribution generated by the pump pulse and coherently sum over the emissions from 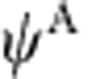 and 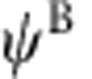



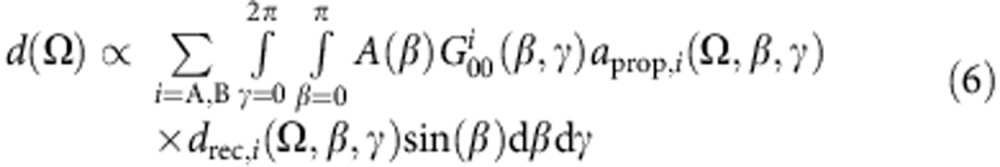


Here *a*_prop,*i*_(Ω,*β*) is the complex amplitude of the recombining continuum electron wave packet and *A*(*β*) is the calculated axis distribution of the molecules.

## Discussion

We now apply this theory (‘full theory' in what follows) to predict the experimental observables. Since we compare ratios, the spectral amplitude of the recombining photoelectron wave packet cancels and we use 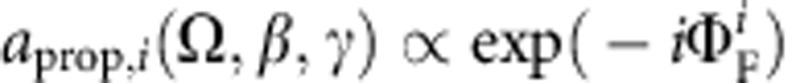. Moreover, frequency-dependent factors relating the induced dipole moment to the intensity^36^ play no role for obtaining ratios. We demonstrate the importance of laser-induced effects by contrasting the predictions of the full theory and the experiments to that of a ‘field-free' theory that neglects the field-induced modification of the orbitals in both ionization and recombination and also neglects the Stark phase 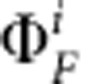.

**Figure 5:**
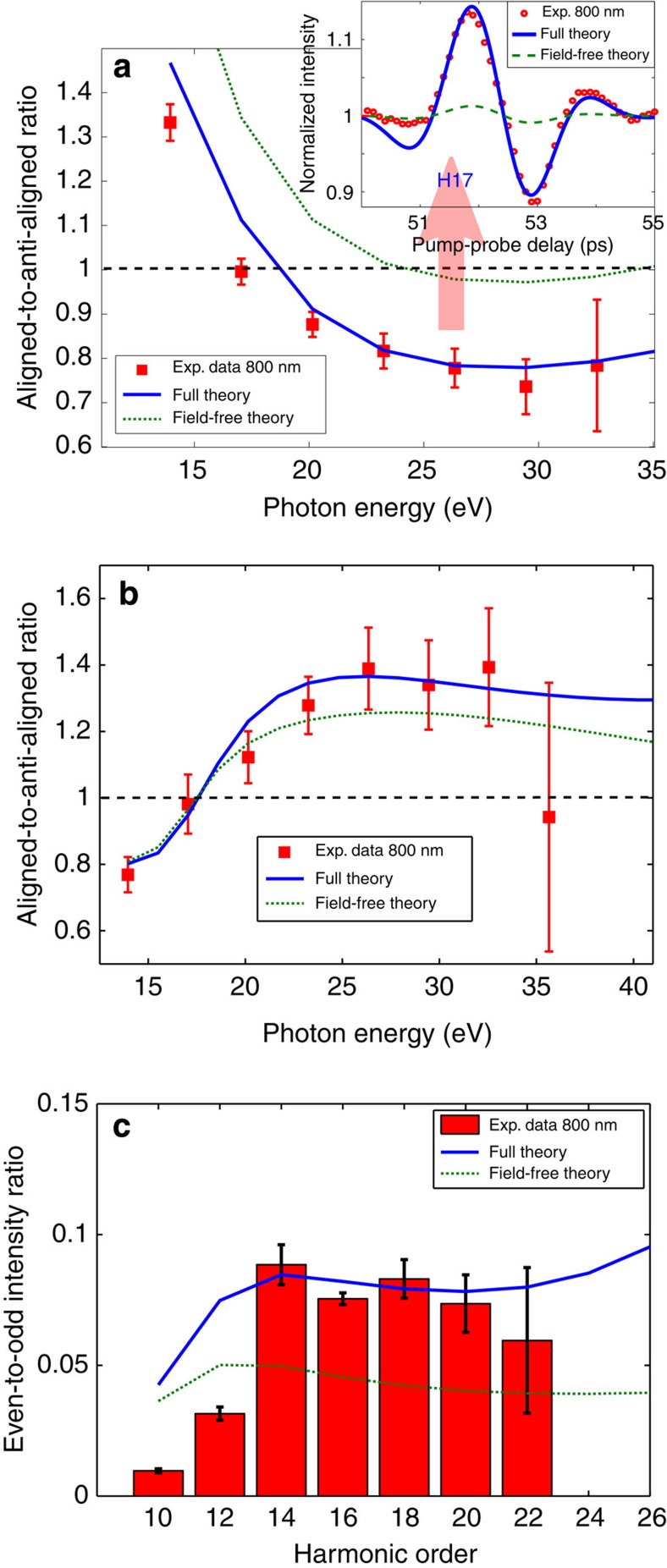
Comparison of high-harmonic intensity ratios with theoretical models. (**a**) Aligned-to-anti-aligned ratios of CH_3_Br compared with the two theories discussed in the text. The inset compares the experimentally observed alignment revival with the predictions of the two theories. (**b**) Same as (**a**) for CH_3_F. (**c**) Even-to-odd ratios for CH_3_F compared with the two theoretical models discussed in the text using 21.5% as degree of orientation. The error bars correspond to twice the s.d. of the measured signal fluctuations.

Figure 5a shows the measured intensity ratio of aligned to anti-aligned CH_3_Br molecules, compared with the field-free and the full theory. Clearly, only the full theory agrees with the experimental observations, whereas the prediction of the field-free theory is qualitatively incorrect in the following way: the reversal of the alignment-induced intensity modulation is observed at H11 (17 eV), whereas the field-free theory predicts the reversal at 24 eV. The field-free theory thus predicts an alignment-induced modulation for H13 and H15 that is opposite to the experimental observation. The full theory, in contrast, correctly and quantitatively predicts the experimental observations.

The field-free theory further predicts an aligned-to-anti-aligned ratio very close to one for H15–H21. Within the signal-to-noise ratio of the experiment, this prediction would correspond to the absence of alignment-induced intensity modulation in H15–H21. This prediction strongly contrasts with the experimental results as is illustrated in the inset of Fig. 5a. This inset further shows that the full theory is in quantitative agreement with the experiment, whereas the field-free theory is qualitatively incorrect because it essentially predicts the absence of alignment-induced modulation.

Figure 5b shows the aligned-to-anti-aligned ratio of CH_3_F. In this case, the predictions of the full and field-free theories do not differ as much as in the case of CH_3_Br. Importantly, the full theory is again in quantitative agreement with the experimental data. This validates our theory further by showing that it does not only quantitatively predict the observations relating to CH_3_Br, but also those of CH_3_F. The deviation of the field-free theory from the experimental data is clearest at H17–H21 (26.3–32.5 eV), where the line corresponding to the full theory lies on top of the experimental data points, whereas the field-free theory consistently underestimates the ratio. The agreement between field-free and full theories at H9–H13 can only be attributed to an accidental cancellation of laser-induced electronic-structure effects in these observables.

Figure 5c shows the even-to-odd ratio of oriented CH_3_F molecules compared with both theories. The field-free theory erroneously predicts an even-to-odd ratio that is almost independent of the harmonic order and fails to reproduce the pronounced increase from H10 to H14. Moreover, the field-free theory strongly underestimates the absolute values of the even-to-odd ratio. In this comparison, the degree of orientation was determined following the approach described in ref. 21 as a single-parameter fit of the full theory to the experimental data which yields 21.5%. Following adjustment of this single parameter, the full theory agrees very well with the measurements. In contrast, the field-free theory predicts an even-to-odd ratio that is much smaller than the observed one. Bringing the field-free theory into remote agreement with the experiment requires a degree of orientation of 29%, much higher than in our calculations that predict 〈cos *θ*〉=0.15±0.01, that is, ∼20% orientation. The remaining deviation of the full theory from the experimental data at H10 and H12 is not entirely unexpected since they lie very close to the ionization threshold of CH_3_F (13.04 eV), where recollision-based theories do not necessarily yield accurate results.

Although Fig. 5 only shows results obtained with 800-nm laser pulses, the theoretical results are independent of the intensity and wavelength of the pulses. This remarkable result follows from two properties: (i) the contribution of the linear Stark effect to the high-harmonic phase is wavelength and intensity independent^37^ and (ii) the contributions from electronically excited states of the cation have been neglected. These two points are strongly supported by the experimentally observed agreement between intensity ratios measured using multiple central wavelengths and intensities (see Fig. 1g,h). Calculations including the first electronically excited state of the cation (that is, the next lower-lying orbital (HOMO-1)) are shown in the Supplementary Fig. 3 and explained in the Supplementary Note 1. These calculations are found to only marginally deviate from the results presented in Fig. 5.

In conclusion, we have observed laser-induced electronic-structure effects in HHS. Generalizing field-free orientation to the non-linear molecules CH_3_F and CH_3_Br, we have observed a reversal of the odd-harmonic signal modulation as a function of the pump–probe delay and a local maximum in the even-to-odd spectral ratio of CH_3_F. All three characteristic features occur at photon energies that are independent of both the intensity and the wavelength of the driving laser field and are therefore not signatures of electronically excited states of the cation. The interpretation of these experimental results required the development of a theory, which explicitly included the modification of the bound electronic states of the molecule. The electric field was found to lift the degeneracy of the E-symmetry electronic ground state of the cation and to create a particular alignment-dependent linear combination of its spatial components. As a consequence of the modified binding energies and wave functions, different sets of linear combinations of a degenerate electronic state become experimentally distinguishable. The contribution of the Stark effect to the phase of high-harmonic emission was also found to be essential in reproducing the experimental results. Although we demonstrated these effects on two symmetric-top molecules, the discussed phenomena are expected to be general in high-harmonic and related strong-field spectroscopies, such as time-resolved studies of chemical dynamics^38^. These effects are of fundamental importance to emerging techniques of attosecond science such as streaking, tunnelling and rescattering^2^,^39^,^40^,^41^,^42^,^43^. They will moreover play a crucial role in extending these techniques to the general class of polar molecules.

## Methods

### Experimental set-up

The experimental set-up consists of an amplified femtosecond titanium:sapphire laser system and a vacuum chamber for generation and spectral characterization of high-order harmonic radiation. The output of the laser system (800 nm, 25 fs and 1 kHz) is either used directly to generate high-harmonic radiation or to pump an optical-parametric amplifier (TOPAS-HE, Light Conversion) tuned to 1,275 or 1,330 nm that generates pulses of ∼40-fs duration. The laser pulses are focused with a spherical (*f*=45 cm) mirror into a pulsed supersonic expansion of 10% CH_3_F in He with 10–20 bar backing pressure. The molecules are either impulsively oriented by a two-colour laser-pulse (800+400 nm, (3±1) × 10^13^ W cm^−2^ peak intensity, 80 fs) or impulsively aligned by a temporally stretched pulse (150 fs) with a lower, non-ionizing intensity ((1±0.5) × 10^13^ W cm^−2^). The alignment/orientation and probe beams are aligned parallel to each other on the spherical mirror with a vertical offset of ∼1 cm and intersect in the molecular beam. The high harmonics generated by the probe beam propagate into an XUV spectrometer consisting of a 120-μm-wide entrance slit, a concave aberration-corrected grating (Shimadzu, 30-002) and a microchannel-plate detector backed with a phosphor screen. A charge-coupled device camera records the spectral images and transfers them to a computer for analysis. More detailed specifications of the set-up and the conditions for generating two-colour laser pulses are given elsewhere^21^.

### Theoretical results

The theoretical results in Fig. 1e,f are obtained by solving the time-dependent Schrödinger equation for a rigid symmetric top using the polarizabilities *α*_|_=17.92 a.u. and *α*_|_=16.29 a.u. (ref. 44) for CH_3_F and *α*_|_=6.890 a.u. and *α*_|_=4.925 a.u. for CH_3_Br from a B3LYP//aug-cc-pVQZ calculation. The molecular-frame photorecombination matrix elements *d*_rec,*i*_(Ω,*β*,*γ*) are calculated using the Schwinger variational method for the electronic continuum implemented in epolyscat^45^,^46^ with orbitals from a Hartree–Fock calculation using the QZVP basis set.

### Calculation of molecular orbitals

The asymptotic behaviour of the structure function *G*_00_^*i*^(*β*,*γ*,*η*) associated with the HOMOs 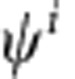 (*i*={A, B}; see Equation (4) of main text) at large *η*=*r*−*z* is the key quantity for the application of the WFAT^29^ for the tunnelling rate. We use a basis-set quantum-chemistry approach using the polarization-consistent (pc) basis sets to obtain the HOMO. These basis sets have been designed and optimized for density functional theory, which have very similar basis-set requirements as HF, and are available in five different quality levels from (unpolarized) double zeta to (polarized) pentuple zeta quality (pc-*n*, *n*=0–4)^47^. It has been shown that these basis sets are capable of reproducing grid-based numerical HF energies for diatomic systems for micro-Hartree accuracy^48^. All basis sets in the present work have been used in their uncontracted forms and variationally optimized with respect to all basis-set exponents. Analytical gradients of the HF energy with respect to basis function exponents have been calculated with the DALTON programme^49^. Basis exponent optimizations have been done using a pseudo-Newton–Raphson method. Starting values were taken from the standard pc-*n* basis sets^47^. In ref. 34 it was shown that this procedure greatly improves the asymptotic behaviour of the wave function at the HF level of theory. All calculations used the experimental geometries of the neutral molecules.

### Determination of Stark-effect coefficients

The Stark-effect coefficients *U* and *V* are obtained in HF//aug-cc-pVQZ calculations with applied static electric fields *F*=0–0.05 a.u. applied along the *x*, *y* or *z* axes defined in Fig. 3. A second-order polynomial is fitted to the HOMO binding energies as a function of the applied field strengths. The *U* and *V* coefficients are taken as the average of the linear coefficients of these fits. This definition of *U* and *V* incorporates the effect of interelectron interaction, in agreement with the many-electron theory^50^. We obtain *U*=0.1606, a.u. and *V*=0.7568, a.u. in the case of CH_3_F and *U*=0.0167, a.u. and *V*=−0.1301, a.u. in the case of CH_3_Br.

## Author contributions

P.M.K. and H.J.W. conceived and designed the experiments. O.I.T., T.M. and L.B.M. developed the theory and calculated the structure factors. P.M.K., D.B., A.R. and J.S. performed the experiments and analysed the data. P.M.K., A.R., J.S. and H.J.W. calculated the photorecombination matrix elements. F.J. calculated the molecular orbitals. C.Z.B. provided computer code for the calculations of rotational dynamics. P.M.K. and H.J.W. wrote the first draft of the manuscript that was finalised by all authors.

## Additional information

**How to cite this article**: Kraus, P.M. *et al.* Observation of laser-induced electronic structure in oriented polyatomic molecules. *Nat. Commun.* 6:7039 doi: 10.1038/ncomms8039 (2015).

## Supplementary Material

Supplementary Information Supplementary Figures 1-3, Supplementary Note 1 and Supplementary References
