# Supplementary material for: Observation of laser-induced electronic structure in oriented polyatomic molecules
Source: Nat Commun. 2015 May 5;6:7039. doi: 10.1038/ncomms8039 (PMC4432593; doi:10.1038/ncomms8039)
Supplement: Supplementary Information — Supplementary Figures 1-3, Supplementary Note 1 and Supplementary References [file ncomms8039-s1.pdf]

Supplementary Figures

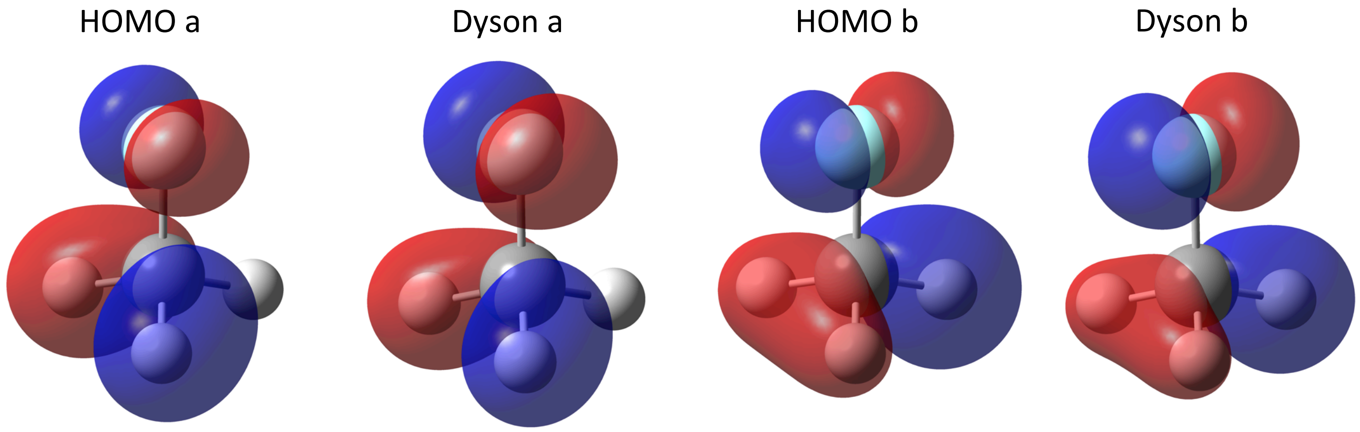

Supplementary Figure 1: Comparison of Hartree-Fock and Dyson orbitals This figure compares the degenerate field-free Hartree-Fock orbitals HOMO a/b obtained from a HF//cc-pVTZ calculation with the Dyson orbitals corresponding to ionisation to the electronic ground state of CH_3F^+ obtained from an EOM-IP-CCSD//cc-pVTZ calculation. The isocontour value for all representations is 0.07. The outline of our theory in the main text is formulated in terms of canonical Hartree-Fock orbitals. Since the electronic ground states of CH_3F^+ and CH_3Br^+ are very well described by a single configuration corresponding to an electron hole in the highest-occupied Hartree-Fock orbital, this is a good approximation. In the recent literature on strong-field spectroscopies, use of Dyson orbitals has become popular. This figure shows that the relevant Hartree-Fock and Dyson orbitals of CH_3F and CH_3Br are barely distinguishable. Hence, use of either type of orbitals leads to virtually identical theoretical results.

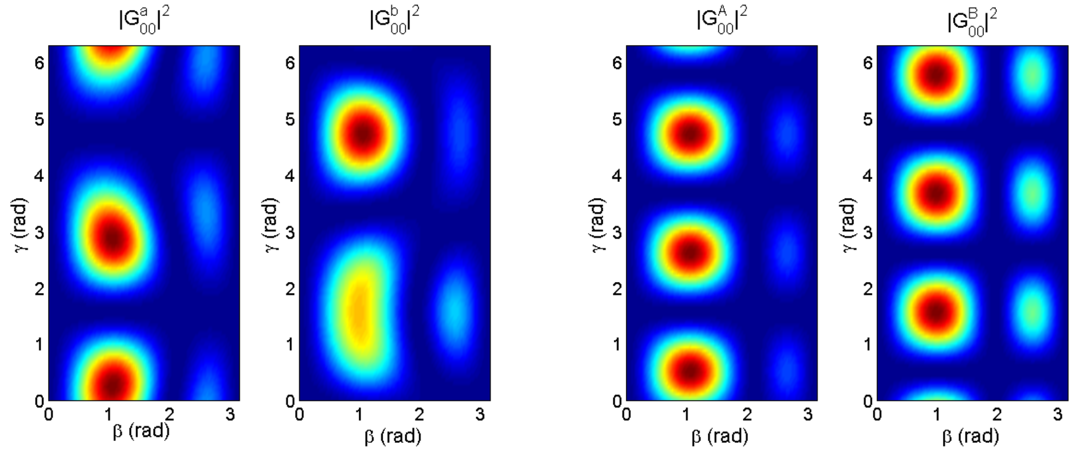

Supplementary Figure 2: Molecular-frame strong-field ionisation rates from the field-free and the full theory The two panels on the left-hand side show the squared structure factors pertaining to the field-free Hartree-Fock HOMOs a/b. The two panels on the right-hand side represent the squared structure factors pertaining to the orbitals A/B. The main text shows that the linear Stark effect on a degenerate electronic state leads to observable differences in high-harmonic intensity ratios. Here we show that strong-field ionisation of molecular samples aligned in three dimensions constitutes another approach to observing the modification of the electronic structure caused by the Stark effect. We note that three-dimensional alignment of CH_3X molecules is not possible within the polarisability interaction, because the polarisability tensor is rotationally symmetric with respect to γ. However, higher-order interactions, such as the hyperpolarisability interaction or strong-field-ionisation depletion could be used to generate a molecular axis distribution that is anisotropic in γ. This figure shows the dependence of the squared structure factors $|G_{00}^{i=\tilde{X};j}|^2$, which are proportional to the strong-field ionisation rates, on the Euler angles β and γ. The predicted molecular-frame ionisation rates from the field-free theory on the left-hand side and the full theory on the right-hand side are substantially different. Molecular-frame measurements of strong-field ionisation rates thus constitute another approach to studying laser-induced electronic structure effects.

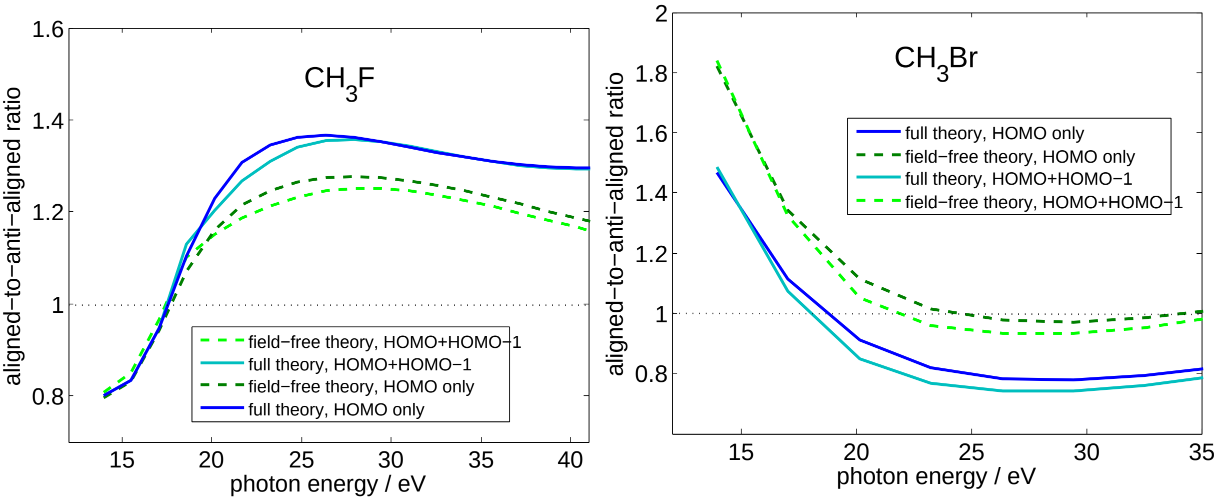

Supplementary Figure 3: Comparison of high-harmonic intensity ratios from calculations including only HOMO or both HOMO and HOMO-1 The full blue lines and the dashed dark-green lines are the same as in Fig. 5 of the main text. The full turquoise and the dashed light-green lines are obtained from calculations based on Eqs. (1) and (2) of this document. Further discussion is given in the Supplementary Note 1 below.

Supplementary Note 1

In this section, we extend the theory formulated in the main text to include electronically-excited states of the cation. The induced dipole moment in the frequency domain is obtained as

$$d(\Omega) \propto \sum_{i=\tilde{\mathrm{X}},\tilde{\mathrm{A}},\ldots} \sum_{j=A,B} \int_{\gamma=0}^{2\pi} \int_{\beta=0}^{\pi} A(\beta) G_{00}^{i,j}(\beta,\gamma) \sqrt{W_{00}^{i}(F)}\, a_{\mathrm{prop},i,j}(\Omega,\beta,\gamma) d_{\mathrm{rec},i,j}(\Omega,\beta,\gamma) \sin(\beta)\, \mathrm{d}\beta \mathrm{d}\gamma, \quad (1)$$

where $\tilde{\mathrm{X}}, \tilde{\mathrm{A}}, \ldots$ represent the ground and electronically-excited states of the molecular cation, $W_{00}^{i}(F)$ is the field factor depending on the laser electric field F and the channel specific (field-free) ionisation potential I_{p}^{i}. The other symbols have the same meaning as in the main text.

As in the main text, we use $a_{\mathrm{prop},i,j}(\Omega,\beta,\gamma) \propto \exp(-\mathrm{i}\Phi_{\mathrm{F}}^{i,j})$ but now include the additional channel-specific accumulated phase arising from different ionisation potentials I_{p}^{i} [1, 2]. We thus obtain (in atomic units):

$$\Phi_{\mathrm{F}}^{i,A/B} = \frac{F_0}{\omega}(\mp U_i \sin(\beta) - V_i \cos(\beta)) \cdot [\sin(\omega t) - \sin(\omega t')] + I_{\mathrm{p}}^{i}(t-t'). \quad (2)$$

The sequence of electronic states of CH_3F^+ and the associated vertical ionisation energies are $\tilde{\mathrm{X}}\ ^2\mathrm{E}$ (13.04 eV), $\tilde{\mathrm{A}}\ ^2\mathrm{A}_1$ (17.20 eV), etc. The electronic states of CH_3Br^+ are $\tilde{\mathrm{X}}\ ^2\mathrm{E}$ (10.54 eV), $\tilde{\mathrm{A}}\ ^2\mathrm{A}_1$ (13.50 eV), etc. The A_1 symmetry of the $\tilde{\mathrm{A}}$ states of CH_3F^+ and CH_3Br^+ leads to $U = 0$. The V coefficients were calculated as described in the methods section of the main text and the values -0.0377 a.u. and -0.6751 a.u. were obtained for CH_3F and CH_3Br, respectively.

The Supplementary Figure 3 shows the results of calculations according to Eqs. (1) and (2) of this document that include both the $\tilde{\mathrm{X}}\ ^2\mathrm{E}$ and the $\tilde{\mathrm{A}}\ ^2\mathrm{A}_1$ states of the cations, i.e. we consider ionisation from HOMO and HOMO-1 in both molecules. This result shows that the contribution from electronically-excited states of the cation is negligible. Hence, these calculations support our experimental conclusion from Fig. 2 of the main text that ionisation to electronically-excited states of the cation plays no significant role in our observables. Hence all results and conclusions of our work are unchanged when contributions from multiple electronic states of the cations are accounted for.

The treatment of the Stark effect in high-harmonic generation involving multiple electronic states deserves a more general additional comment. The theory outlined in this article only includes the linear Stark effect. This is a good approximation when the separation of the electronic states is much larger than their Stark shift. When this is not the

case, higher-order contributions to the Stark effect need to be considered, which is straightforward to implement.

Supplementary References

[1] Kanai, T., Takahashi, E. J., Nabekawa, Y. & Midorikawa, K. Destructive interference during high harmonic generation in mixed gases. *Phys. Rev. Lett.* **98**, 153904 (2007).
[2] Kraus, P. M. & Wörner, H. J. Time-resolved high-harmonic spectroscopy of valence electron dynamics. *Chemical Physics* **414**, 32 – 44 (2013).
